# Short-Term Outcomes in Patients Undergoing Virtual/Ghost Ileostomy or Defunctioning Ileostomy after Anterior Resection of the Rectum: A Meta-Analysis

**DOI:** 10.3390/jcm12113607

**Published:** 2023-05-23

**Authors:** Maurizio Zizzo, Andrea Morini, Magda Zanelli, David Tumiati, Francesca Sanguedolce, Andrea Palicelli, Federica Mereu, Stefano Ascani, Massimiliano Fabozzi

**Affiliations:** 1 Surgical Oncology Unit, Azienda Unità Sanitaria Locale-IRCCS di Reggio Emilia, 42123 Reggio Emilia, Italy; 2 Pathology Unit, Azienda Unità Sanitaria Locale-IRCCS di Reggio Emilia, 42123 Reggio Emilia, Italy; 3 Pathology Unit, Azienda Ospedaliero-Universitaria, Ospedali Riuniti di Foggia, 71122 Foggia, Italy; 4 Hematology Unit, CREO, Azienda Ospedaliera di Perugia, University of Perugia, 06129 Perugia, Italy; 5 Pathology Unit, Azienda Ospedaliera S. Maria di Terni, University of Perugia, 05100 Terni, Italy

**Keywords:** anterior rectal resection, rectum, ileostomy, colorectal cancer, surgery, outcomes

## Abstract

Background and Objectives: Anterior rectal resection (ARR) represents one of the most frequently performed methods in colorectal surgery, mainly carried out for rectal cancer (RC) treatment. Defunctioning ileostomy (DI) has long been chosen as a method to “protect” colorectal or coloanal anastomosis after ARR. However, DI does not rule out risks of more or less serious complications. A proximal intra-abdominal closed-loop ileostomy, the so-called virtual/ghost ileostomy (VI/GI), could limit the number of DIs and the associated morbidity. Materials and Methods: We performed a systematic review following the Preferred Reporting Items for Systematic Reviews and Meta-Analyzes (PRISMA) guidelines. Meta-analysis was performed by use of RevMan [Computer program] Version 5.4. Results: The five included comparative studies (VI/GI or DI) covering an approximately 20-year study period (2008–2021). All included studies were observational ones and originated from European countries. Meta-analysis indicated VI/GI as significantly associated with lower short-term morbidity rates related to VI/GI or DI after primary surgery (RR: 0.21, 95% CI: 0.07–0.64, *p* = 0.006), fewer dehydration (RR: 0.17, 95% CI: 0.04–0.75, *p* = 0.02) and ileus episodes after primary surgery (RR: 0.20, 95% CI: 0.05–0.77, *p* = 0.02), fewer readmissions after primary surgery (RR: 0.17, 95% CI: 0.07–0.43, *p* = 0.0002) and readmissions after primary surgery plus stoma closure surgery (RR: 0.14, 95% CI: 0.06–0.30, *p* < 0.00001) than the DI group. On the contrary, no differences were identified in terms of AL after primary surgery, short-term morbidity after primary surgery, major complications (CD ≥ III) after primary surgery and length of hospital stay after primary surgery. *Conclusions*: Given the significant biases among meta-analyzed studies (small overall sample size and the small number of events analyzed, in particular), our results require careful interpretation. Further randomized, possibly multi-center trials may be of paramount importance in confirming our results.

## 1. Introduction

In May 1948, the President of Mayo Clinic General Surgery, Claude F. Dixon, first introduced the concept and the promising short- and long-term results of the anterior rectal resection (ARR) technique for tumors of the proximal rectum and distal sigmoid colon [1,2,3]. The following years witnessed a prompt spread of the aforementioned technique that also turned out to be the gold standard for treating lower-middle rectal cancer (RC) [3].

In 1975, Samuel N. Fain described the use of a circular stapler of Russian origin to perform colorectal anastomosis during ARR [1,4]. Later, successful outcomes by Mark M. Ravitch also paved the way for the rapid spread of stapling tools in the United States [1,5].

To date, ARR represents one of the most frequently performed methods in colorectal surgery [1,3]. It is mainly carried out for RC treatment, although it also finds application in the treatment of various other major conditions involving the rectum, both malignant (i.e., ovarian cancer) and benign (i.e., endometriosis) ones.

The early detection and correct treatment of morbidity related to this type of intervention, among which anastomotic leakage (AL) stands out above all, represents the most important aspects related to the postoperative management of ARR patients [6,7]. In the last decades, scientifically reported AL incidence has not significantly changed, although continued improvements in both stapled and manual sutures, preoperative patient evaluation as well as surgical technique have been recorded [6,7]. Over the years, several risk factors for AL have been identified [8,9]. However, AL remains an undesirable complication that can also seriously impact cancer patients’ prognosis [7]. Colorectal surgery AL incidence recorded a 2.8–30% rate, out of which 75% of cases occurred in rectal anastomosis with a 2–16.4% mortality rate and a 20–35% morbidity rate [7].

Defunctioning ileostomy (DI) for colorectal or coloanal anastomosis has long been chosen as a method to “protect” anastomosis from insults to the repair process [10,11,12,13]. Although several studies have recorded homogeneous AL rates in deviated patients and undeviating ones, the consequences of pelvic sepsis recorded a considerable reduction with diversion [10,11,12,13].

However, performing DI does not rule out risks of more or less serious complications that are related not only to stoma creation (e.g., electrolyte imbalances, wound infections) but also to stoma closure (e.g., suture dehiscence) [10,11,12,13]. Thus, Sacchi et al. first carried out a proximal intra-abdominal closed-loop ileostomy, the so-called virtual/ghost ileostomy (VI/GI), the rationale for which was to selectively perform a DI when early signs of AL were detected [14,15]. Such a process is made easier by fixing the distal ileum to the abdominal wall, by use of an external silastic tube at the time of rectal resection [14,15]. Subsequently, in case the leakage does not require emergency laparotomy, DI can be performed through a trephine incision [14,15]. This approach aims to limit the number of DIs and the associated morbidity [14,15,16].

Our meta-analysis aimed at providing updated evidence from a comparison between short-term outcomes among patients who have undergone ARR with VI/GI or DI.

## 2. Materials and Methods

The meta-analysis was performed following the Preferred Reporting Items for Systematic Reviews and Meta-Analyses (PRISMA) statement and guidelines [17].

Moreover, a review protocol was entered into the PROSPERO database (CRD42022369057).

As our meta-analysis was based on previously published studies and there was no addition of original patient population data, approval by the ethics committee and informed patient consent were not required.

### 2.1. Search Strategy

PubMed/MEDLINE, Scopus, Web of Science (Science and Social Science Citation Index), Embase and Cochrane Library (Cochrane Database of Systematic Reviews, Cochrane Central Register of Controlled Trials-CENTRAL) databases were used to identify articles of interest.

The combination of non-MeSH/MeSH terms was as follows:

#### 2.1.1. PubMed/MEDLINE

(((virtual[Title/Abstract]) AND (ileostomy[Title/Abstract])) OR (ghost[Title/Abstract])) AND (ileostomy[Title/Abstract]).

#### 2.1.2. Scopus

(TITLE-ABS-KEY (virtual) AND TITLE-ABS-KEY (ileostomy) OR TITLE-ABS-KEY (ghost) AND TITLE-ABS-KEY (ileostomy)).

#### 2.1.3. Web of Science

(((Topic = (virtual)) AND Topic = (ileostomy)) OR Topic = (ghost)) AND Topic = (ileostomy).

#### 2.1.4. Embase

(virtual:ti,ab,kw OR ghost:ti,ab,kw) AND ileostomy:ti,ab,kw.

#### 2.1.5. Cochrane Library

Title Abstract Keyword AND ileostomy in Title Abstract Keyword OR ghost in Title Abstract Keyword AND ileostomy in Title Abstract Keyword—(word variations were searched).

Final analysis was carried out on 29 September 2022.

Moreover, the reference lists of included studies and relevant reviews were manually searched.

### 2.2. Inclusion Criteria

Comparative population studies (case series, case-control studies, cohort studies, controlled clinical trials and randomized clinical trials) concerning adult patients (over 18 years of age) undergoing ARR plus VI/GI or DI were included.

Abstracts, posters, letters to the editor, editorials, case reports and previously published systematic reviews and/or meta-analyses were ruled out, although previously published systematic reviews or meta-analyses were taken into account in order to identify comparative studies left out through our systematic search.

Due to the limited data found during the first unsystematic search, our systematic search ruled out restrictions in terms of language, date of issue and surgically treated primary pathology.

### 2.3. Outcomes

Primary outcomes included AL after primary surgery, short-term morbidity after primary surgery, major complications (Clavien-Dindo or CD ≥ III) after primary surgery and short-term morbidity related to VI/GI or DI after primary surgery, while secondary outcomes included dehydration after primary surgery, ileus after primary surgery, readmissions after primary surgery, readmissions after primary surgery plus stoma closure surgery and length of hospital stay after primary surgery.

### 2.4. Data Extraction

Papers were selected and identified by two independent reviewers (M.Zi. and A.M.) based on title, abstracts, keywords and full texts. The following data were collected from the included papers:

- Demographic data [Author’s surname and year of publication; study type; study country; study period; population size, gender and age; body mass index (BMI); American Society of Anesthesiologists’ (ASA) score];
- Surgical data [primary surgical pathology; surgical procedure; surgical approach; preoperative treatment; VI/GI removal timing];
- Outcomes data [AL after primary surgery, short-term morbidity after primary surgery, major complications (CD ≥ III) after primary surgery, short-term morbidity related to VI/GI or DI after primary surgery, dehydration after primary surgery, ileus after primary surgery, readmissions after primary surgery, readmissions after primary surgery plus stoma closure surgery and length of hospital stay after primary surgery].

All collected results were eventually reviewed by a third independent reviewer (M.F.).

### 2.5. Quality Assessment

For an accurate quality assessment of the different included studies, two independent reviewers analyzed them using RoB 2 and ROBINS-I tools [18,19].

Version 2 of the Cochrane Risk-of-Bias tool for randomized trials (RoB 2) was recommended for assessing the risk of bias in randomized trials [18]. It included a fixed set of bias domains that were focused on different aspects of study design, conduct and reporting [18]. Each domain involved a series of questions (“reporting questions”) aimed at collecting data on study features that were relevant to the risk of bias [18]. A proposal for bias risk from each domain was generated by an algorithm, based on answers to the questions reported [18]. The ratings for risk of bias were “Low”, “High” or “Some Concerns” [18].

The ROBINS-I tool was developed to assess the risk of bias in non-randomized studies, and compared health outcomes of two or more interventions [19]. In order to obtain an assessment of the risk, reporting questions were used that had a substantial factual nature and aimed at easing judgment on the risk of bias [19]. Answers to the reporting questions provided a framework for domain-level judgments on the risk of bias, which then served as a basis for an overall judgment on the risk of bias in a special outcome [19]. The ratings for risk of bias judgments were “Low Risk”, “Moderate Risk”, “Severe Risk” and “Critical Risk”, keeping in mind that “Low risk” meant the risk of bias in a high-quality randomized study [19]. Only in exceptional cases will a non-randomized study be rated as low risk of bias due to confounding variables [19].

### 2.6. Statistical Analysis

Our meta-analysis was performed using “Review Manager (RevMan) [Computer program] Version 5.4. The Cochrane Collaboration, 2020” [20]. For dichotomous outcomes, risk ratios (RRs) and corresponding 95% confidence intervals (CIs) were computed according to the Mantel–Haenszel (MH) method. For continuous outcomes, weighted mean differences (WMDs) and corresponding 95% CIs were computed using the inverse variance method. In terms of the lack of mean or standard deviation (SD) for an end-point, this was obtained from reported median range, or interquartile range (IQR), if provided.

I^2^ statistics were used to assess statistical heterogeneity; <25, 25–50 and >50% I^2^ values were classified as follows: low, moderate and high. Due to the heterogeneity of malignant diseases and patient features, in addition to discrepancies in surgical approaches and adopted methods, a random effects model was used as the default in all statistical analyses. Statistical significance was set at *p* < 0.05. Moreover, a subgroup analysis stratified by primary surgical pathology was carried out.

## 3. Results

### 3.1. Search Results

**Figure 1:**
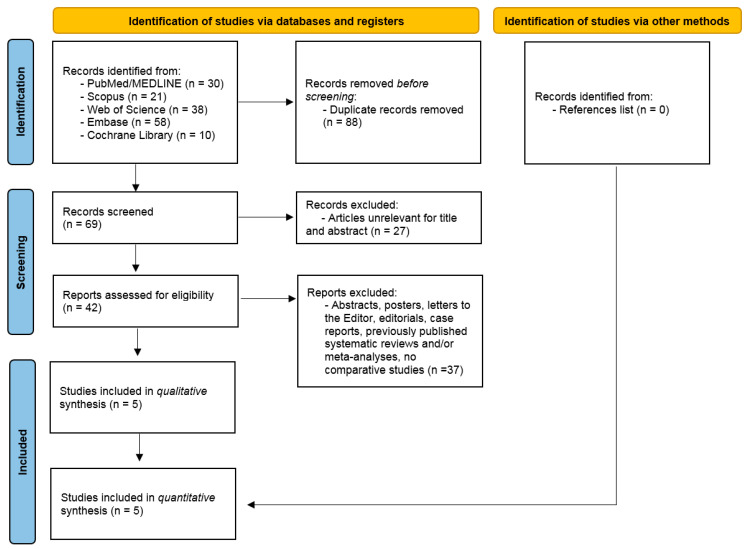
PRISMA flow chart of the literature search.

According to the final literature search of 29 September 2022, 157 potentially interesting studies were found (PubMed/MEDLINE: 30 records; Scopus: 21 records; Web of Science: 38 records; Embase: 58 records; Cochrane Library: 10 records) (Figure 1). Although our analysis covered all 157 studies, 88 out of 157 studies were ruled out as duplicate publications. Furthermore, 27 turned out to be irrelevant for title and abstract, while 42 full texts were considered eligible. Due to the ruling out of thirty-seven studies for not complying with inclusion criteria, five comparative studies underwent qualitative and quantitative synthesis [21,22,23,24,25]. No additional records were found through other sources (references list).

### 3.2. Quality of Studies

According to ROBINS-I, most non-randomized studies showed serious overall bias [22,23,25], except for Gullà et al. (moderate) [21] and Zenger et al. (critical) [24] (see Supplementary Materials, Table S1). The RoB2 tool was not employed due to a lack of identification of randomized trials.

### 3.3. Study Characteristics

Table 1 shows the study characteristics. The five studies identified through the systematic search were all observational ones. Out of them, three had a retrospective design and two had a prospective one. They all stemmed from Europe and covered a nearly 20-year observational period (between 2008 and 2021).

The pooled population included 342 patients: 47% (161) underwent ARR and VI/GI, while 53% (181) underwent ARR and DI.

### 3.4. Population Characteristics

Table 1 and Table 2 show the general and surgical-specific features of the analyzed populations. The mean/median BMI of populations under analysis ranged between 21 and 27.4. Furthermore, 84% of patients (286/342) had ASA score I-II and 73% (250/342) had rectal cancer as the primary disease.

Considering the surgical-specific data, 73% of patients (190/260; four of the five included studies) underwent low ARR (LARR), with an equal distribution between open approaches and laparoscopic approaches. Most patients received preoperative treatment with chemotherapy (CT) and/or radiotherapy (RT) (71%, 200/281; four of the five included studies).

### 3.5. Meta-Analyses Results

#### 3.5.1. Anastomotic Leakage after Primary Surgery

**Figure 2:**
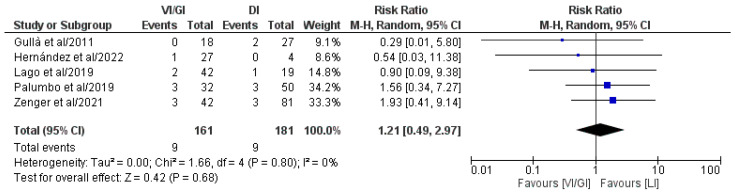
Forest plot comparing anastomotic leakage after primary surgery between the VI/GI and DI groups. CI, confidence interval; M-H, Mantel–Haenszel [21,22,23,24,25].

Overall, all five of the included studies (342 patients: VI/GI 161, DI 181) recorded an anastomotic leakage rate following primary surgery (Figure 2) [21,22,23,24,25]. Meta-analysis of the pooled results showed that the anastomotic leakage rate after primary surgery (RR: 1.21, 95% CI: 0.49–2.97, *p* = 0.68) did not record statistically significant differences between the two groups. Detected heterogeneity was low, although statistically negligible (I^2^ = 0%, *p* = 0.80).

#### 3.5.2. Short-Term Morbidity after Primary Surgery

**Figure 3:**
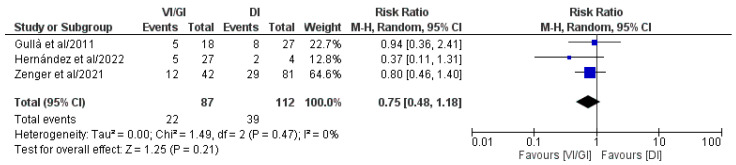
Forest plot comparing short-term morbidity after primary surgery between the VI/GI and DI groups. CI, confidence interval; M-H, Mantel–Haenszel [21,24,25].

Overall, three out of the five included studies (199 patients: VI/GI 87, DI 112) recorded short-term morbidity following primary surgery (Figure 3) [21,24,25]. Meta-analysis of pooled results showed that short-term morbidity following primary surgery (RR: 0.75, 95% CI: 0.48–1.18, *p* = 0.21) did not record statistically significant differences between the two groups. Detected heterogeneity was low, although statistically negligible (I^2^ = 0%, *p* = 0.47).

#### 3.5.3. Major Complications (CD ≥ III) after Primary Surgery

**Figure 4:**
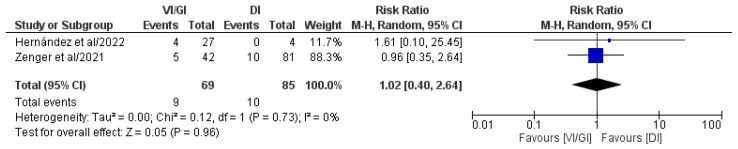
Forest plot comparing major complications (CD ≥ III) after primary surgery between the VI/GI and DI groups. CI, confidence interval; M-H, Mantel–Haenszel [24,25].

Overall, two out of the five included studies (154 patients: VI/GI 69, DI 85) reported major complications (CD ≥ III) after primary surgery (Figure 4) [24,25]. Meta-analysis of the pooled results showed that major complications (CD ≥ III) after primary surgery (RR: 1.02, 95% CI: 0.40–2.64, *p* = 0.96) did not record statistically significant differences between the two groups. Detected heterogeneity was low, although statistically negligible (I^2^ = 0%, *p* = 0.73).

#### 3.5.4. Short-Term Morbidity Related to VI/GI or DI after Primary Surgery

**Figure 5:**
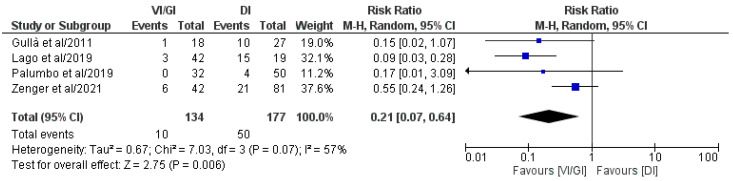
Forest plot comparing short-term morbidity related to VI/GI or DI after primary surgery between the VI/GI and DI groups. CI, confidence interval; M-H, Mantel–Haenszel [21,22,23,24].

Overall, four out of the five included studies (311 patients: VI/GI 134, DI 177) reported short-term morbidity related to VI/GI or DI after primary surgery (Figure 5) [21,22,23,24]. Meta-analysis of the pooled results showed that, compared to the DI group, the VI/GI group recorded statistically significant lower short-term morbidity related to VI/GI or DI after primary surgery (RR: 0.21, 95% CI: 0.07–0.64, *p* = 0.006). Detected heterogeneity was high, although statistically negligible (I^2^ = 57%, *p* = 0.07).

#### 3.5.5. Dehydration after Primary Surgery

**Figure 6:**
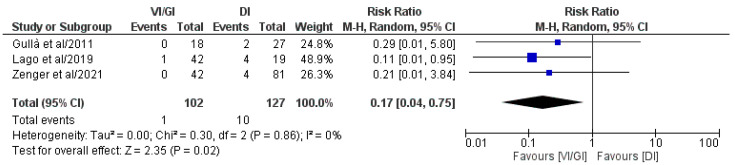
Forest plot comparing the dehydration rate after primary surgery between the VI/GI and DI groups. CI, confidence interval; M-H, Mantel–Haenszel [21,22,24].

Overall, three out of the five included studies (comprising 229 patients: VI/GI 102, DI 127) reported dehydration rates after primary surgery (Figure 6) [21,22,24]. Meta-analysis of the pooled results showed that the VI/GI group recorded a statistically significant lower dehydration rate after primary surgery (RR: 0.17, 95% CI: 0.04–0.75, *p* = 0.02) compared to the DI group. The detected heterogeneity was low, although statistically negligible (I^2^ = 0%, *p* = 0.86).

#### 3.5.6. Ileus after Primary Surgery

**Figure 7:**
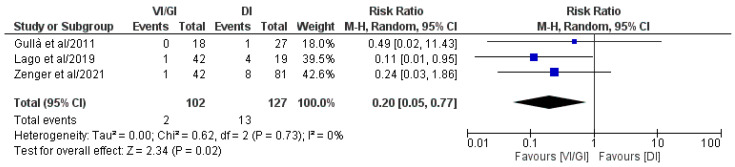
Forest plot comparing the ileus rate after primary surgery between the VI/GI and DI groups. CI, confidence interval; M-H, Mantel–Haenszel [21,22,24].

Overall, three out of the five included studies (229 patients: VI/GI 102, DI 127) reported the ileus rate after primary surgery (Figure 7) [21,22,24]. Meta-analysis of the pooled results showed that the VI/GI group recorded a statistically significant lower ileus rate after primary surgery (RR: 0.20, 95% CI: 0.05–0.77, *p* = 0.02) compared to the DI group. Detected heterogeneity was low, although statistically negligible (I^2^ = 0%, *p* = 0.73).

#### 3.5.7. Readmissions after Primary Surgery

**Figure 8:**
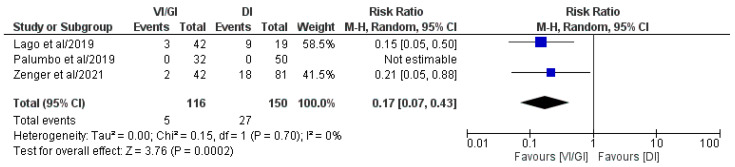
Forest plot comparing readmissions after primary surgery between the VI/GI and DI groups. CI, confidence interval; M-H, Mantel–Haenszel [22,23,24].

Overall, three out of the five included studies (266 patients: VI/GI 116, DI 150) reported readmissions after primary surgery (Figure 8) [22,23,24]. Meta-analysis of the pooled results showed that the VI/GI group showed statistically significant lower readmissions after primary surgery (RR: 0.17, 95% CI: 0.07–0.43, *p* = 0.0002) compared to the DI group. The detected heterogeneity was low, although statistically negligible (I^2^ = 0%, *p* = 0.70).

#### 3.5.8. Readmissions after Primary Surgery Plus Stoma Closure Surgery

**Figure 9:**
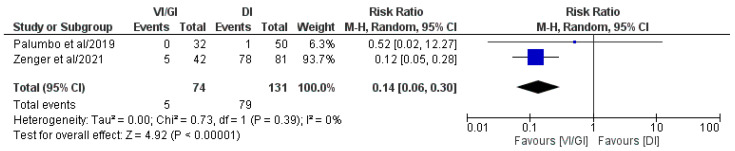
Forest plot comparing readmissions after primary surgery plus stoma closure surgery between the VI/GI and DI groups. CI, confidence interval; M-H, Mantel–Haenszel [23,24].

Overall, two out of the five included studies (205 patients: VI/GI 74, DI 131) reported readmissions after primary surgery plus stoma closure surgery (Figure 9) [23,24]. Meta-analysis of the pooled results showed that the VI/GI group recorded statistically significant lower readmission rates after primary surgery plus stoma closure surgery (RR: 0.14, 95% CI: 0.06–0.30, *p* < 0.00001) compared to the DI group. Detected heterogeneity was low, although statistically negligible (I^2^ = 0%, *p* = 0.39).

#### 3.5.9. Length of Hospital Stay after Primary Surgery

**Figure 10:**
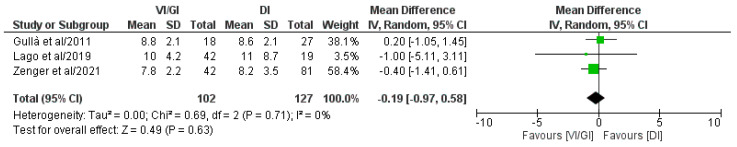
Forest plot comparing the length of hospital stay after primary surgery between the VI/GI and DI groups. SD, Standard Deviation; CI, confidence interval [21,22,24].

Overall, four out of the five included studies reported the length of hospital stay after primary surgery [21,22,24,25]. However, the study by Hernández et al. was excluded due to a lack of data needed to perform the meta-analysis (229 patients: VI/GI 102, DI 127) (Figure 10) [21,22,24]. Meta-analysis of the pooled results showed that the length of hospital stay after primary surgery (MD: −0.19, 95% CI: −0.97–0.58, *p* = 0.63) did not record statistically significant differences between the two groups. Detected heterogeneity was low, although statistically negligible (I^2^ = 0%, *p* = 0.39).

#### 3.5.10. Subgroup Analysis

Subgroup analysis was carried out according to discrepancies in the study designs. In particular, we analyzed different outcomes only considering studies that had rectal cancer as the primary disease. Our subgroup analysis confirmed the outcomes of the pooled analysis: a statistically significant lower readmission rate after primary surgery (RR: 0.21, 95% CI: 0.05–0.88, *p* < 0.03, I^2^ = not applicable) and a readmission rate after primary surgery plus stoma closure surgery (RR: 0.14, 95% CI: 0.06–0.30, *p* < 0.00001, I^2^ = 0%) in the VI/GI group, as well as a lower short-term morbidity related to VI/GI or DI after primary surgery (RR: 0.43, 95% CI: 0.20–0.89, *p* < 0.02, I^2^ = 0%) in the VI/GI group (see Supplementary Materials, Figures S1–S9).

#### 3.5.11. Publication Bias

As our meta-analysis included five studies, we did not carry out an analysis of publication bias. Indeed, according to the Cochrane Handbook for Systematic Reviews of Interventions (Version 5.1.0), tests for funnel plot asymmetry should only be carried out in meta-analyses of at least 10 studies [26]. As a matter of fact, fewer studies reduced the power of tests to identify the case from real asymmetry [26].

## 4. Discussion

The present meta-analysis first investigated short-term outcomes of comparative studies on patient populations undergoing ARR plus VI/GI or DI. Our study on a pooled 342-patient population (161 patients treated with ARR plus VI/GI and 181 patients treated with ARR plus DI) highlighted how VI/GI brought several statistically significant advantages when compared to DI, such as lower short-term morbidity rates related to VI/GI or DI after primary surgery, fewer dehydration and ileus episodes after primary surgery, fewer readmissions after primary surgery and readmissions after primary surgery plus stoma closure surgery. On the contrary, no statistically significant differences were identified concerning AL after primary surgery, short-term morbidity after primary surgery and major complications (CD ≥ III) after primary surgery, representing the main outcomes of our study.

As previously mentioned in our introduction, AL is one of the most feared complications in colorectal surgery and is almost always one of the main outcomes examined in comparative studies of both benign and malignant colorectal surgery [7]. Although this outcome is one of the main study aims when investigating colorectal surgery, there was no international definition that can standardize the different international surgical experiences and make the published results less heterogeneous until at least 2010 [6,7]. Later, the International Study Group of Rectal Cancer (ISGRC) introduced AL definition and grading following ARR, which represents the most exhaustive and accepted method even today [27]. In particular, AL is described as a communication between intra- and extraluminal compartments, stemming from a defective integrity of the intestinal wall during colorectal or coloanal anastomosis [27]. Due to a similar clinical impact, a leak originating from the suture or staple line of the neorectal reservoir (e.g., J-pouch or transverse coloplasty) should also be considered an anastomotic leak [27]. Moreover, consensus recommendations suggest that any pelvic abscess near anastomosis should be considered a leak [27].

Over the years, many mainly clinical observational studies have tried to identify colorectal AL risk factors by classifying them as local or general factors, pre, intra or post-operative factors or modifiable or non-modifiable factors [9,28,29]. Preoperative factors, such as male gender, ASA score > 2, the Comprehensive Complication Index (CCI) ≥ 3 (strictly related to diabetes mellitus, cardiovascular disease, renal failure, chronic pulmonary obstruction) and a history of radiotherapy, are the most reported non-modifiable factors [9]. Moreover, non-modifiable intraoperative factors such as tumor distance from the anal margin, emergency surgery, operating time and surgeon skills have been highlighted [9]. A systematic review by Wallace et al. aimed to summarize all of the modifiable risk factors of colorectal AL from available Cochrane systematic reviews [8]. Author analysis identified 20 modifiable risk factors [8]. Out of them, three were statistically related to colorectal AL: low (versus high) surgeon’s operative volume (RR = 0.68); handsewn (versus stapled) ileocolic anastomosis (RR = 0.41); no ostomy (versus defunctioning ostomy) in ARR for RC (RR = 0.32) [8].

The identification of risk factors in AL can help surgeons choose tailored approaches in their clinical practice. As previously mentioned, many studies have identified different risk factors, although it is still very difficult to predict AL onset in individual patients [6,7,8,9,28,29]. Pre- or intraoperative evaluations leading to anastomosis and/or stoma remain difficult. Several leak scores have been developed to help surgeons make an unbiased assessment of AL risks and choose surgical management approaches [30,31,32,33,34]. Nevertheless, the decision still appears discretionary and dependent on the surgeon’s mind, as highlighted by all the studies included in the present meta-analysis.

In principle, defunctioning ostomy protects colorectal/coloanal anastomosis through a loop transverse colostomy or loop ileostomy, although both the conventional use and effective role of a protective stoma following colorectal surgery still encourages strong debate in the scientific literature [7]. Phan et al.’s 2019 meta-analysis only included prospective RCTs comparing populations undergoing ARR with or without ostomy for low RC [35]. Authors analyzed an overall 892-patient population (460 patients with ostomy and 432 patients without ostomy) and identified a significant reduction in AL and reoperation rates in the ostomy group, in absence of statistically significant differences in other crucial outcomes (perioperative mortality, wound infection, postoperative bleeding, small bowel obstruction, peritonitis, pulmonary infection, urinary tract infection and permanent ostomy) [35]. In 2022, Emile et al. introduced outcomes from their meta-analysis, the design and scope of which was comparable to Phan et al.’s study [36]. The authors analyzed a 946-patient population (489 patients with ostomy and 457 patients without ostomy) [36]. The ostomy group recorded a lower rate of statistically significant overall complications, ALs, abscesses and reoperations, reporting no differences in perioperative mortality, small bowel obstruction and surgical site infection (SSI) [36]. Both of the above meta-analyses supported the creation of ostomy in ARR for low-medium RC [35,36].

Further controversy exists regarding the most optimal type of ostomy to perform following ARR: an ileostomy or a colostomy [7]. In recent years, meta-analyses comparing colostomy versus ileostomy in patients undergoing ARR have been published with the aim of assessing short-term assets and liabilities [7]. As concerned outcomes related to stoma formation (high-output stoma, stoma prolapse, skin irritation, skin retraction, parastomal hernia, parastomal sepsis and overall complication rate), a meta-analysis by Gavrilidis et al. found that the stomal prolapse rate was significantly higher in the colostomy group, while a much higher stomal output was recorded in the ileostomy group [37]. On the contrary, concerning outcomes related to ostomy closure (wound infection, time to stoma reversal, operative time, anastomotic leakage/fistula, length of hospital stay, overall complications, mortality) wound infections and incisional hernias were significantly less frequent in the ileostomy group [37]. Both meta-analyses carried out by Chudner et al. and Du et al. largely confirmed the outcomes determined by Gavriilidis et al. [38,39]. The colostomy group recorded higher overall morbidity rates after stoma creation and closure, higher levels of stomal prolapse, SSI and parastomal hernia, all following stoma creation [38,39]. The ileostomy group witnessed increased ileus and SSI rates, both after closure of the stoma [38,39]. Taking into account the results of the aforementioned meta-analyses, the superiority of one method over the other remains uncertain [37,38,39].

Dehydration related to stomal output is one of the main complications related to ileostomy [40]. In a recent meta-analysis by Boruck et al., which aimed to identify incidence of dehydration and dehydration-related morbidity in patient populations undergoing colorectal resection for RC with or without DI, a 1.2–42.8% between-study rate and a 9% pooled probability were recorded [41]. When comparative studies paralleled DI and defunctioning colostomy (DC), the relative risk of dehydration was 3.31; while, by comparing DI and no ostomy, it was 3.42, and by comparing DI and DC with no ostomy, it was 3.37 [41]. As underlined by the scientific literature, acute kidney injury (AKI) and related hospital readmissions are the main clinical consequences of dehydration following ileostomy [41]. However, even ileostomised patients showing no symptoms and signs of AKI may experience progressive deterioration of renal function [42]. In fact, recent studies have shown that 25% of patients develop chronic kidney injury (CKI) at 2 years [42]. Our meta-analysis confirmed that, due to the absence of stomal output, the rate of dehydration turned out to be significantly lower in patients with VI/GI (functionally without ostomy) than in patients with DI.

Readmissions after the creation of a DI are very frequent, while having a bad impact on a patient’s convalescence [43]. Reasons for readmissions are mainly stoma-related, such as dehydration, stomal outlet obstruction, peristomal skin lesions, anastomotic leakage and overall postoperative complications, such as infections and thromboembolic events [43]. Recent meta-analysis by Vogel et al. aimed to assess the prevalence of readmissions related to dehydration, following DI creation [43]. The authors reported an overall 20% readmission rate within 30 days with dehydration as the leading cause, occurring in one-third of ileostomy patients readmitted to hospital (6%) [43]. Concerning the data related to dehydration outcomes, our meta-analysis confirmed the data already reported in the scientific literature. Indeed, our results confirmed that the VI/GI group showed a significantly lower rate of readmissions, both following primary surgery and after primary surgery plus stoma closure surgery.

Overall, an assessment of our results did not show statistically significant differences between the VI/GI group (patients to be functionally considered without ostomy) and the DI group concerning AL after primary surgery, short-term morbidity after primary surgery, major complications after primary surgery and the length of hospital stay after primary surgery. Conversely, all other outcomes taken into account were significantly better in the VI/GI group. Therefore, the abovementioned results might suggest that VI/GI creation is preferable to DI creation in selected cases.

The current meta-analysis did have several limitations. No randomized controlled study was identified, except for observational studies, and they were all retrospective studies and lacked propensity score matching analysis. Both the number of included studies and the number of patients enrolled in the included populations were low. These populations were heterogeneous and concerned general characteristics, the primary pathology treated (benign/malignant, colorectal malignant/ovarian malignant) and the adopted surgical techniques (ARR/LARR) and approaches (laparoscopic/open). Moreover, although some included studies reported the definition and grading of AL of the ISGRC, no homogeneity was found. Furthermore, all of the included studies reported that whether to perform a VI/GI or a DI or not was entirely related to the surgeon’s choice.

Despite the aforementioned shortcomings, significant strengths not identified in previous meta-analyses [16,44] were highlighted in our study. In the absence of randomized controlled trials or propensity score matching observational trials, by only including comparative (double-arm) studies, our meta-analysis reached the highest level of evidence on this topic. As in previous studies [44], the integration of both comparative and non-comparative (single-arm) studies could lead to non-negligible increased bias. Unlike other meta-analyses that include any type of article [44], by only including full, peer-reviewed articles, we made the best choice in setting up a meta-analysis, as it is common for the raw data of a study to be presented via abstracts, letters, posters or conference proceedings. Such data are often later revised and corrected by authors, both during the pre-submission review and in the course of the peer-review process. Moreover, our meta-analysis included a significantly higher number of outcomes than those examined by other systematic reviews/meta-analyses [44]. Finally, our meta-analysis collected comparative studies related to whatever the type of primary surgical disease, and not only to rectal cancer [44], the results of which were also introduced by a peculiar subgroup analysis.

As mentioned above, a subgroup analysis was carried out that only focused on patient populations undergoing ARR for RC (three out of five studies [21,23,24]), in order to reduce the bias related to the primary surgical disease. In principle, subgroup analysis corroborated the results related to the pooled population: statistically significant lower readmissions after primary surgery and readmissions following primary surgery plus stoma closure surgery in the VI/GI group, in addition to a lower short-term morbidity related to VI/GI or DI after primary surgery.

## 5. Conclusions

Our meta-analysis of comparative studies concerning adult patients undergoing ARR plus VI/GI or DI showed that the VI/GI group had a lower short-term morbidity rate related to VI/GI or DI after primary surgery, fewer dehydration and ileus episodes after primary surgery, fewer readmissions after primary surgery and readmissions after primary surgery plus stoma closure surgery than the DI group. On the contrary, following primary surgery, no differences were identified in terms of AL, short-term morbidity after primary surgery, major complications (CD ≥ III) after primary surgery and the length of hospital stay after primary surgery.

However, given the significant biases among meta-analyzed studies, the small overall sample size and the small number of events analyzed, our results require careful interpretation. Indeed, in order to confirm our results and reach an appropriate and uniform patient selection, well-designed randomized controlled trials, possibly multicenter ones, are strongly recommended.

## Figures and Tables

**Table 1:** Study and population characteristics.

Authors/Year	Study Type	Study Country	Study Period	Group	Patient Population, *n*	Gender, *n*		Age (Years)	BMI (kg/m^2^)	ASA Score, *n*			
						Male	Female			I	II	III	IV
Gullà et al./2011 [21]	Prospective	Italy	2008–2009	VI/GI	18	10	8	72 (43–86)	22.7 (6.5)	11	6	1	0
				DI	27	15	12	73 (48–88)	23.6 (8.2)	16	9	2	0
Lago et al./2019 [22]	Retrospective	Spain	2010–2018	VI/GI	42	n/a	n/a	54.5 ± 10 (32–77)	24.8 ± 2.5 (15–35)	39		3	
				DI	19	n/a	n/a	60.9 ± 8.8 (39–76)	25.1 ± 6 (16–42)	12		7	
Palumbo et al./2019 [23]	Retrospective	Italy	2015–2017	VI/GI	32	14	18	65.68 ± 10.01	23.97 ± 3.44	28	45	9	0
				DI	50	34	16	68.77 ± 11.01	25.22 ± 4.41				
Zenger et al./2021 [24]	Retrospective	Turkey	2010–2019	VI/GI	42	24	18	61 ± 11	27.4 ± 4.6	8	21	13	0
				DI	81	52	29	60 ± 11	26.6 ± 3.8	9	51	21	0
Hernández et al./2022 [25]	Prospective	Germany	2019–2021	VI/GI	27	0	27	31 (28–34)	24 (22–27)	16	11	0	0
				DI	4	0	4	31 (29–36)	21(19–32)	1	3	0	0

*n* = number; BMI = Body Mass Index; ASA = American Society of Anesthesiologists; VI/GI = virtual/ghost ileostomy; DI = defunctioning ileostomy; n/a = not available.

**Table 2:** Surgical-specific characteristics.

Authors/Year	Group	Patient Population, *n*	Surgical Pathology, *n*			Surgical Procedure, *n*		Surgical Approach, *n*		Preoperative Treatment, *n*				VI Removal, Days
			Endometriosis	Ovarian Cancer	Rectal Cancer	ARR	LARR	Laparoscopy	Open	CT	RT	CRT	No	
Gullà et al./2011 [21]	VI/GI	18	0	0	18	0	18	0	18	0	0	18	0	10–15 after primary surgery
	DI	27	0	0	27	0	27	0	27	0	0	27	0	_
Lago et al./2019 [22]	VI/GI	42	0	42	0	36	6	0	42	n/a	0	0	n/a	14 days after discharge
	DI	19	0	19	0	18	1	0	19	n/a	0	0	n/a	_
Palumbo et al./2019 [23]	VI/GI	32	0	0	32	n/a	n/a	n/a	n/a	0	0	32	0	8 after primary surgery
	DI	50	0	0	50	n/a	n/a	n/a	n/a	0	0	50	0	_
Zenger et al./2021 [24]	VI/GI	42	0	0	42	0	42	35	7	0	0	10	32	9–12 after primary surgery
	DI	81	0	0	81	0	81	64	17	0	17	46	18	_
Hernández et al./2022 [25]	VI/GI	27	27	0	0	14	13	27	0	0	0	0	27	after bowel function recovery or at discharge
	DI	4	4	0	0	2	2	4	0	0	0	0	4	_

*n* = number; ARR = anterior rectal resection; LARR = low anterior rectal resection; CT = chemotherapy; RT = radiotherapy; CRT = chemoradiotherapy; VI/GI = virtual/ghost ileostomy; DI = defunctioning ileostomy; n/a = not available.

## Data Availability

The data presented in this study are available on request from the corresponding author.

## Supplementary Materials

The following supporting information can be downloaded at: https://www.mdpi.com/article/10.3390/jcm12113607/s1, Table S1 Retrospective studies evaluated using ROBINS-I.; Figure S1. Forest plot comparing anastomotic leakage after primary surgery between the VI/GI and DI groups [RC subgroups]. CI, confidence interval; M-H, Mantel–Haenszel.; Figure S2. Forest plot comparing short-term morbidity after primary surgery between the VI/GI and DI groups [RC subgroups]. CI, confidence interval; M-H, Mantel–Haenszel.; Figure S3. Forest plot comparing major complications (CD ≥ III) after primary surgery between the VI/GI and DI groups [RC subgroups]. CI, confidence interval; M-H, Mantel–Haenszel.; Figure S4. Forest plot comparing short-term morbidity related to VI/GI or DI after primary surgery between the VI/GI and DI groups [RC subgroups]. CI, confidence interval; M-H, Mantel–Haenszel.; Figure S5. Forest plot comparing dehydration rate after primary surgery between the VI/GI and DI groups [RC subgroups]. CI, confidence interval; M-H, Mantel–Haenszel.; Figure S6. Forest plot comparing ileus rate after primary surgery between the VI/GI and DI groups [RC subgroups]. CI, confidence interval; M-H, Mantel–Haenszel.; Figure S7. Forest plot comparing readmissions after primary surgery between the VI/GI and DI groups [RC subgroups]. CI, confidence interval; M-H, Mantel–Haenszel.; Figure S8. Forest plot comparing readmissions after primary surgery plus stoma closure surgery be-tween the VI/GI and DI groups [RC subgroups]. CI, confidence interval; M-H, Mantel–Haenszel; Figure S9. Forest plot comparing length of hospital stay after primary surgery between the VI/GI and DI groups [RC subgroups]. SD, Standard Deviation; CI, confidence interval.
