# Supplementary material for: Short-Term Outcomes in Patients Undergoing Virtual/Ghost Ileostomy or Defunctioning Ileostomy after Anterior Resection of the Rectum: A Meta-Analysis
Source: J Clin Med. 2023 May 23;12(11):3607. doi: 10.3390/jcm12113607 (PMC10253561; doi:10.3390/jcm12113607)
Supplement: Supplementary file 1 [file jcm-12-03607-s001.zip › jcm-2357813-supplementary.pdf]

Supplementary material

Tables S1. Retrospective studies evaluated using ROBINS-I.

		Gullà et al/2011 [21]	Lago et al/2019 [22]	Palumbo et al/2019 [23]	Zenger et al/2021 [24]	Hernández et al/2022 [25]
Preintervention	Confounding	Moderate[1]	Serious[1,2]	Serious[1,3]	Critical [1,4,5]	Serious[1,2]
	Selection bias	Low	Low	Low	Low	Low
Intraintervention	Classification of interventions	Low	No information[6]	No information[6]	Low	No information[6]
Postintervention	Intended interventions	Low	Low	Low	Low	Low
	Missing data	Low	Low	Low	Low	Low
	Measurement of outcomes	Low	Low	Low	Low	Low
	Reported results	Low	Low	Low	Low	Low
Overall bias		Moderate	Serious	Serious	Critical	Serious

[1] Eligibility for a certain type of surgical procedure was determined by the surgeon.
[2] Surgical procedures varied among patients.
[3] No detailed information regarding surgical procedure and surgical approach was reported.
[4] Surgical approaches varied among patients.
[5] Preoperative treatments varied among patients.
[6] No information was reported about whether start of follow up and start of intervention coincide.

Study or Subgroup	VI/GI Events	VI/GI Total	DI Events	DI Total	Weight	Risk Ratio M-H, Random, 95% CI
Gullà et al/2011	0	18	2	27	11.9%	0.29 [0.01, 5.80]
Palumbo et al/2019	3	32	3	50	44.6%	1.56 [0.34, 7.27]
Zenger et al/2021	3	42	3	81	43.5%	1.93 [0.41, 9.14]
**Total (95% CI)**		**92**		**158**	**100.0%**	**1.40 [0.50, 3.92]**
Total events	6		8			

Heterogeneity: Tau² = 0.00; Chi² = 1.26, df = 2 (P = 0.53); I² = 0%
Test for overall effect: Z = 0.65 (P = 0.52)

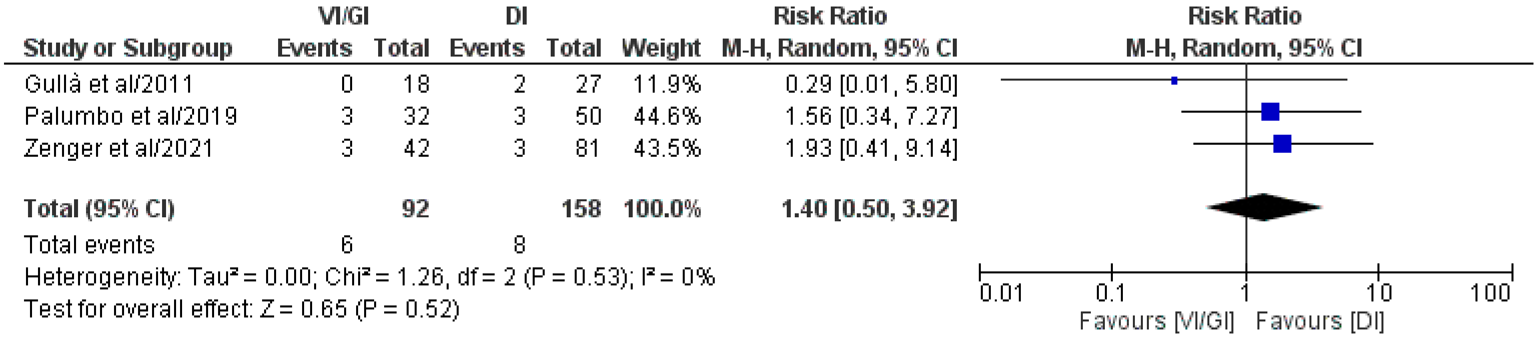

Figure S1. Forest plot comparing anastomotic leakage after primary surgery between the VI/GI and DI groups [RC subgroups]. CI, confidence interval; M-H, Mantel–Haenszel. [21,23,24]

Study or Subgroup	VI/GI Events	VI/GI Total	DI Events	DI Total	Weight	Risk Ratio M-H, Random, 95% CI
Gullà et al/2011	5	18	8	27	26.0%	0.94 [0.36, 2.41]
Zenger et al/2021	12	42	29	81	74.0%	0.80 [0.46, 1.40]
**Total (95% CI)**		**60**		**108**	**100.0%**	**0.83 [0.51, 1.35]**
Total events	17		37			

Heterogeneity: Tau² = 0.00; Chi² = 0.08, df = 1 (P = 0.77); I² = 0%
Test for overall effect: Z = 0.75 (P = 0.45)

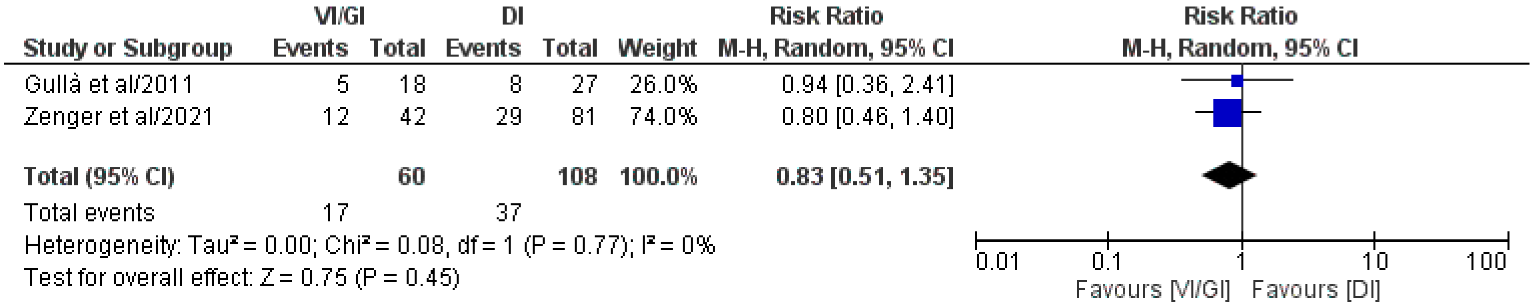

Figure S2. Forest plot comparing short-term morbidity after primary surgery between the VI/GI and DI groups [RC subgroups]. CI, confidence interval; M-H, Mantel–Haenszel. [21,24]

Study or Subgroup	VI/GI Events	VI/GI Total	DI Events	DI Total	Weight	Risk Ratio M-H, Random, 95% CI
Zenger et al/2021	5	42	10	81	100.0%	0.96 [0.35, 2.64]
**Total (95% CI)**		**42**		**81**	**100.0%**	**0.96 [0.35, 2.64]**
Total events	5		10			

Heterogeneity: Not applicable
Test for overall effect: Z = 0.07 (P = 0.94)

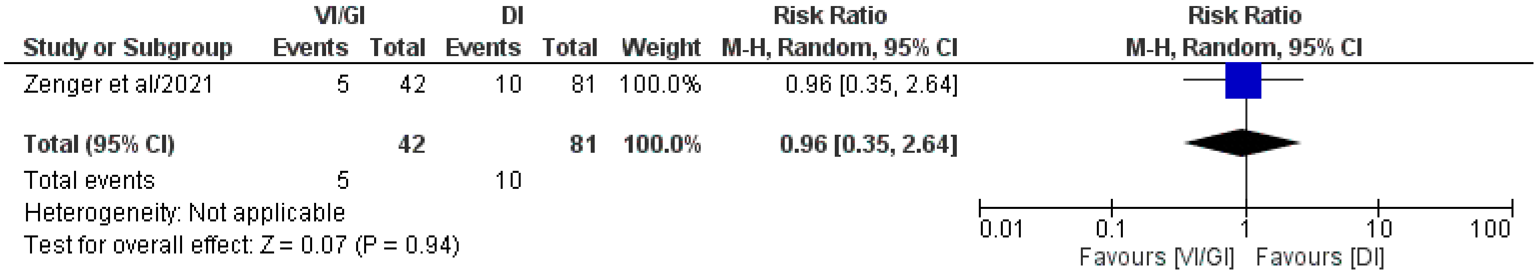

Figure S3. Forest plot comparing major complications (CD≥III) after primary surgery between the VI/GI and DI groups [RC subgroups]. CI, confidence interval; M-H, Mantel–Haenszel. [24]

Study or Subgroup	VI/GI Events	VI/GI Total	DI Events	DI Total	Weight	Risk Ratio M-H, Random, 95% CI
Gullà et al/2011	1	18	10	27	14.0%	0.15 [0.02, 1.07]
Palumbo et al/2019	0	32	4	50	6.5%	0.17 [0.01, 3.09]
Zenger et al/2021	6	42	21	81	79.4%	0.55 [0.24, 1.26]
**Total (95% CI)**		**92**		**158**	**100.0%**	**0.43 [0.20, 0.89]**
Total events	7		35			

Heterogeneity: Tau² = 0.00; Chi² = 1.94, df = 2 (P = 0.38); I² = 0%
Test for overall effect: Z = 2.27 (P = 0.02)

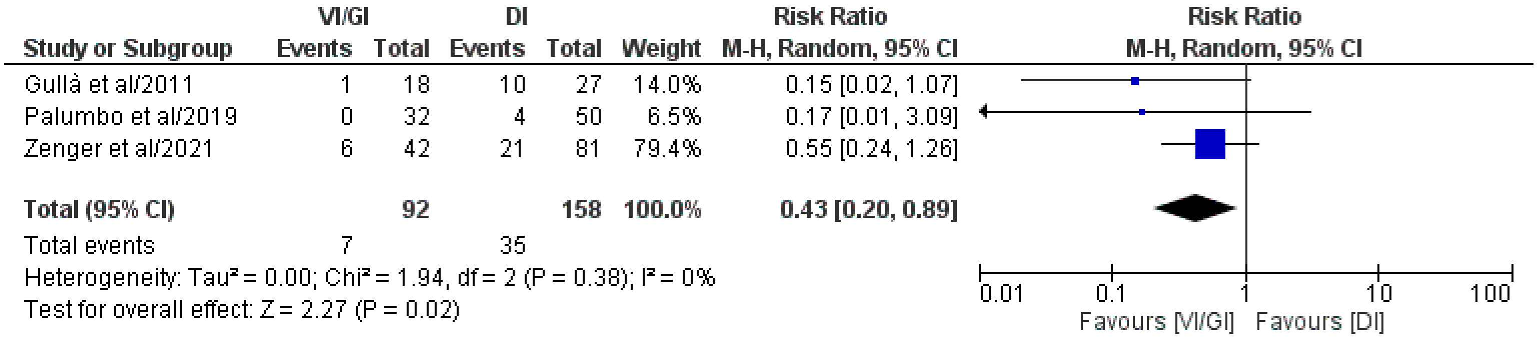

Figure S4. Forest plot comparing short-term morbidity related to VI/GI or DI after primary surgery between the VI/GI and DI groups [RC subgroups]. CI, confidence interval; M-H, Mantel–Haenszel. [21,23,24]

Study or Subgroup	VI/GI Events	VI/GI Total	DI Events	DI Total	Weight	Risk Ratio M-H, Random, 95% CI
Gullà et al/2011	0	18	2	27	48.6%	0.29 [0.01, 5.80]
Zenger et al/2021	0	42	4	81	51.4%	0.21 [0.01, 3.84]
**Total (95% CI)**		**60**		**108**	**100.0%**	**0.25 [0.03, 1.99]**
Total events	0		6			

Heterogeneity: Tau² = 0.00; Chi² = 0.02, df = 1 (P = 0.88); I² = 0%
Test for overall effect: Z = 1.31 (P = 0.19)

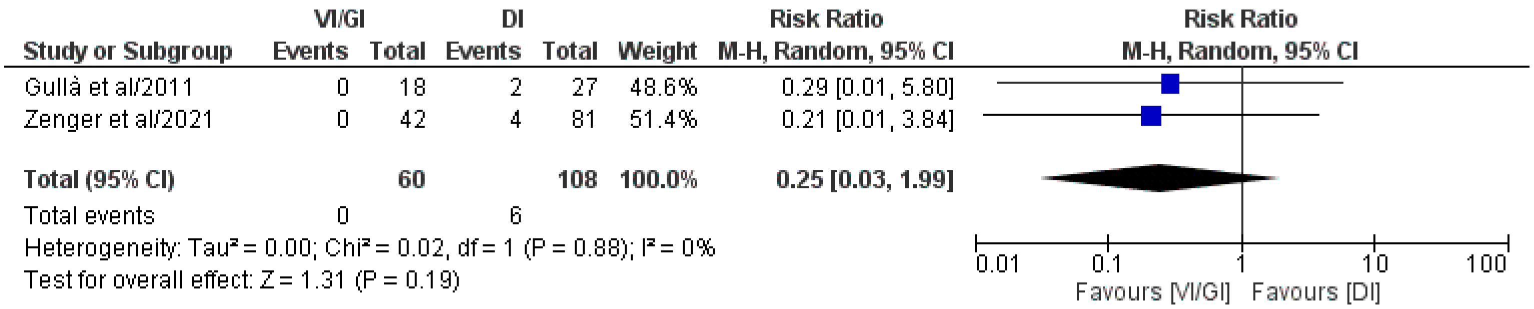

Figure S5. Forest plot comparing dehydration rate after primary surgery between the VI/GI and DI groups [RC subgroups]. CI, confidence interval; M-H, Mantel–Haenszel. [21,24]

Study or Subgroup	VI/GI Events	VI/GI Total	DI Events	DI Total	Weight	Risk Ratio M-H, Random, 95% CI
Gullà et al/2011	0	18	1	27	29.7%	0.49 [0.02, 11.43]
Zenger et al/2021	1	42	8	81	70.3%	0.24 [0.03, 1.86]
**Total (95% CI)**		**60**		**108**	**100.0%**	**0.30 [0.05, 1.65]**
Total events	1		9			

Heterogeneity: Tau² = 0.00; Chi² = 0.14, df = 1 (P = 0.71); I² = 0%
Test for overall effect: Z = 1.38 (P = 0.17)

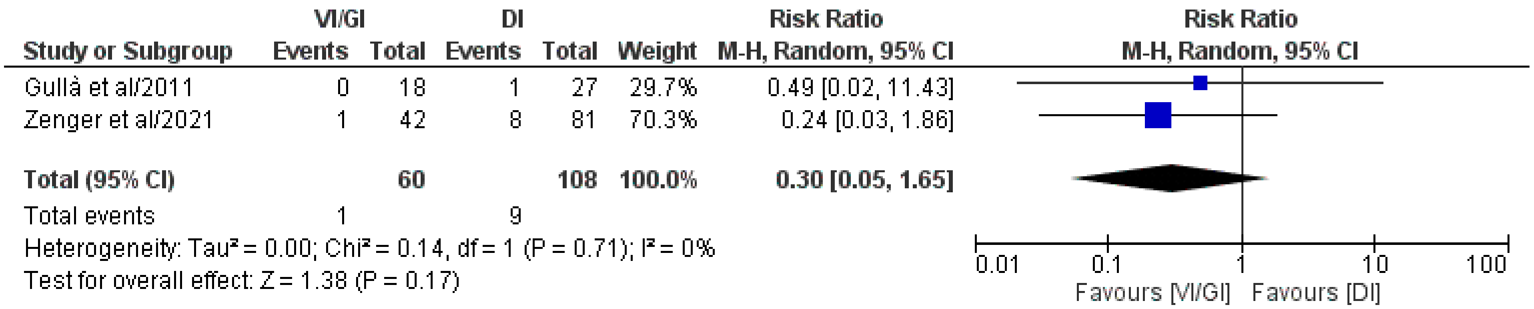

Figure S6. Forest plot comparing ileus rate after primary surgery between the VI/GI and DI groups [RC subgroups]. CI, confidence interval; M-H, Mantel–Haenszel. [21,24]

Study or Subgroup	VI/GI Events	VI/GI Total	DI Events	DI Total	Weight	Risk Ratio M-H, Random, 95% CI
Palumbo et al/2019	0	32	0	50		Not estimable
Zenger et al/2021	2	42	18	81	100.0%	0.21 [0.05, 0.88]
**Total (95% CI)**		**74**		**131**	**100.0%**	**0.21 [0.05, 0.88]**
Total events	2		18			

Heterogeneity: Not applicable
Test for overall effect: Z = 2.14 (P = 0.03)

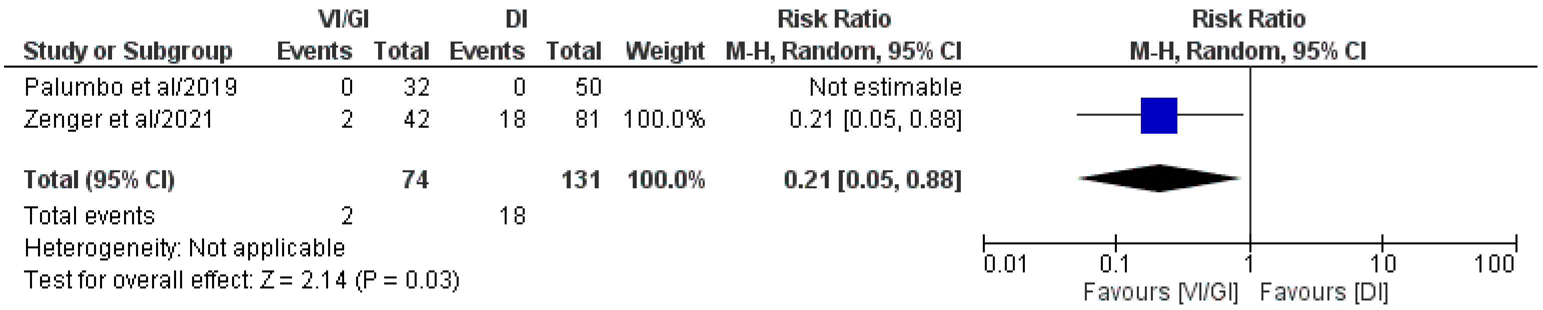

Figure S7. Forest plot comparing readmissions after primary surgery between the VI/GI and DI groups [RC subgroups]. CI, confidence interval; M-H, Mantel–Haenszel. [23,24]

Study or Subgroup	VI/GI Events	VI/GI Total	DI Events	DI Total	Weight	Risk Ratio M-H, Random, 95% CI
Palumbo et al/2019	0	32	1	50	6.3%	0.52 [0.02, 12.27]
Zenger et al/2021	5	42	78	81	93.7%	0.12 [0.05, 0.28]
**Total (95% CI)**		**74**		**131**	**100.0%**	**0.14 [0.06, 0.30]**
Total events	5		79			

Heterogeneity: Tau² = 0.00; Chi² = 0.73, df = 1 (P = 0.39); I² = 0%
Test for overall effect: Z = 4.92 (P < 0.00001)

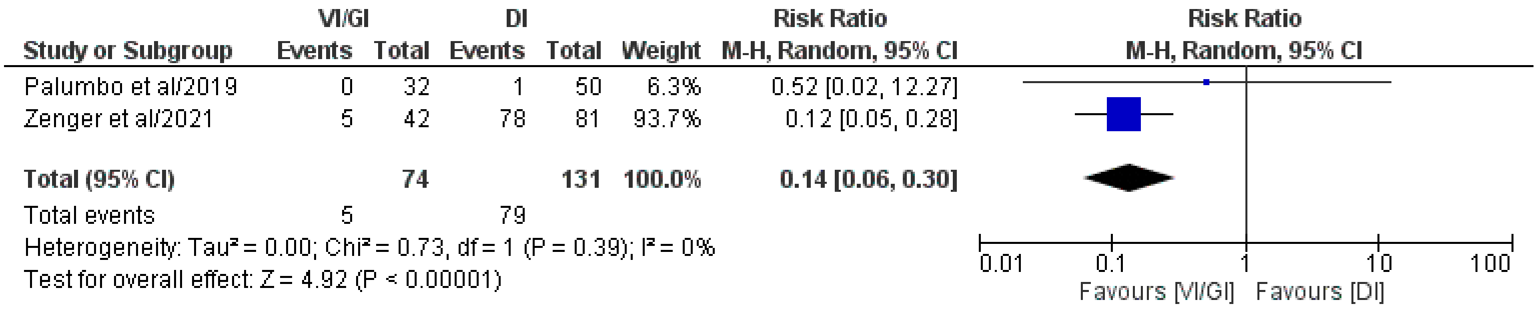

Figure S8. Forest plot comparing readmissions after primary surgery plus stoma closure surgery between the VI/GI and DI groups [RC subgroups]. CI, confidence interval; M-H, Mantel–Haenszel. [23,24]

Study or Subgroup	VI/GI Mean	VI/GI SD	VI/GI Total	DI Mean	DI SD	DI Total	Weight	Mean Difference IV, Random, 95% CI
Gullà et al/2011	8.8	2.1	18	8.6	2.1	27	39.5%	0.20 [-1.05, 1.45]
Zenger et al/2021	7.8	2.2	42	8.2	3.5	81	60.5%	-0.40 [-1.41, 0.61]
**Total (95% CI)**			**60**			**108**	**100.0%**	**-0.16 [-0.95, 0.62]**

Heterogeneity: Tau² = 0.00; Chi² = 0.53, df = 1 (P = 0.47); I² = 0%
Test for overall effect: Z = 0.41 (P = 0.68)

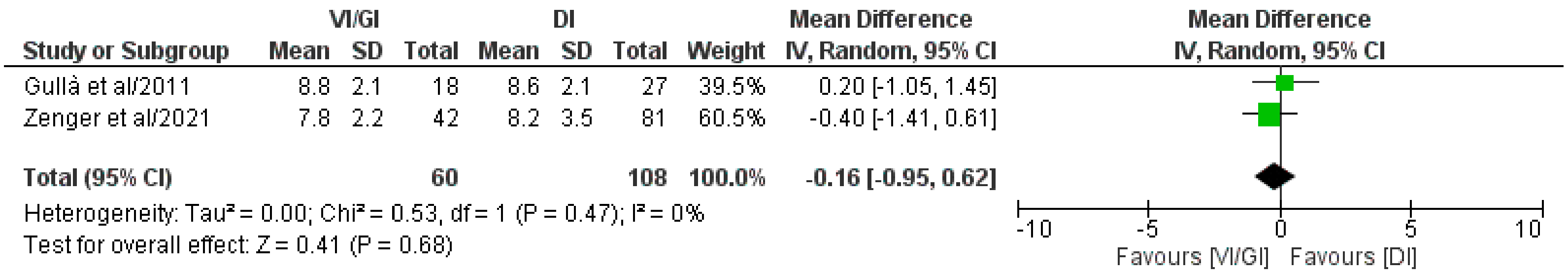

Figure S9. Forest plot comparing length of hospital stay after primary surgery between the VI/GI and DI groups [RC subgroups]. SD, Standard Deviation; CI, confidence interval. [21,24]
